# Nanofibrous Chitosan-Polyethylene Oxide Engineered Scaffolds: A Comparative Study between Simulated Structural Characteristics and Cells Viability

**DOI:** 10.1155/2014/438065

**Published:** 2014-06-04

**Authors:** Mohammad Kazemi Pilehrood, Mandana Dilamian, Mina Mirian, Hojjat Sadeghi-Aliabadi, Laleh Maleknia, Pertti Nousiainen, Ali Harlin

**Affiliations:** ^1^Department of Materials Science, Tampere University of Technology, P.O. Box 589, 33101 Tampere, Finland; ^2^Department of Textile Engineering, Islamic Azad University South Tehran Branch, P.O. Box 11365-4435, Tehran, Iran; ^3^Department of Pharmaceutical Chemistry, School of Pharmacy, Isfahan University of Medical Sciences, P.O. Box 81745-359, Isfahan, Iran; ^4^VTT Technical Research Centre of Finland, P.O. Box 1000, 02044 VTT, Finland

## Abstract

3D nanofibrous chitosan-polyethylene oxide (PEO) scaffolds were fabricated by electrospinning at different processing parameters. The structural characteristics, such as pore size, overall porosity, pore interconnectivity, and scaffold percolative efficiency (SPE), were simulated by a robust image analysis. Mouse fibroblast cells (L929) were cultured in RPMI for 2 days in the presence of various samples of nanofibrous chitosan/PEO scaffolds. Cell attachments and corresponding mean viability were enhanced from 50% to 110% compared to that belonging to a control even at packed morphologies of scaffolds constituted from pores with nanoscale diameter. To elucidate the correlation between structural characteristics within the depth of the scaffolds' profile and cell viability, a comparative analysis was proposed. This analysis revealed that larger fiber diameters and pore sizes can enhance cell viability. On the contrary, increasing the other structural elements such as overall porosity and interconnectivity due to a simultaneous reduction in fiber diameter and pore size through the electrospinning process can reduce the viability of cells. In addition, it was found that manipulation of the processing parameters in electrospinning can compensate for the effects of packed morphologies of nanofibrous scaffolds and can thus potentially improve the infiltration and viability of cells.

## 1. Introduction

**Figure 1:**
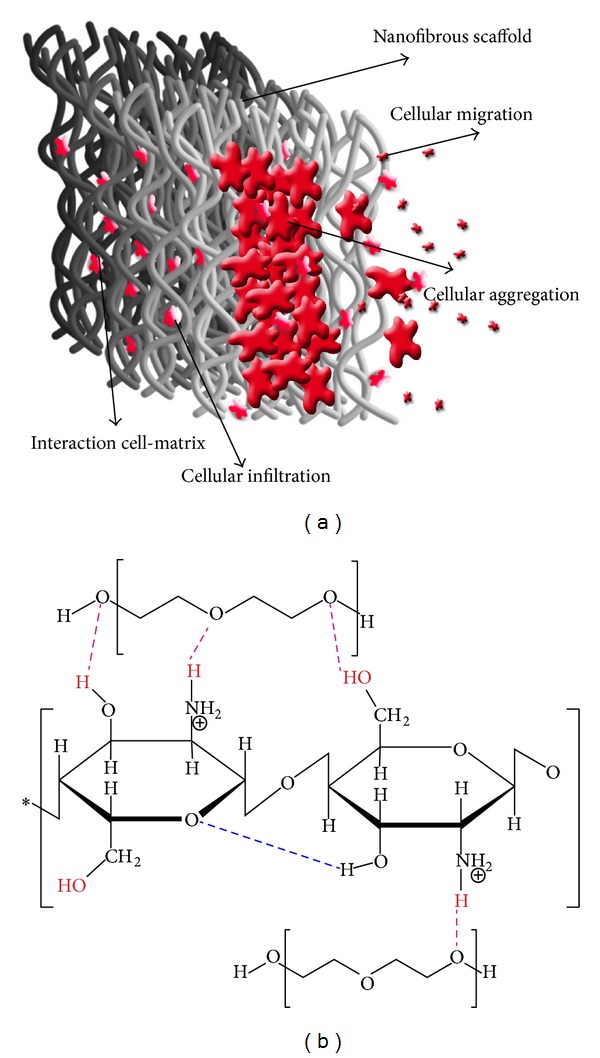
(a) Schematic illustration of interactions between cells and nanofibrous scaffold (matrix). (b) Chemical structure of chitosan/PEO polymer chains and their hydrogen bonding.

In tissue regeneration, many attempts have been made to explore the material properties and processing methods possessing the highest biomimicry with native tissues. Implantations of 3D fibrous scaffolds can mimic the extracellular matrix (ECM)consisting of proteoglycans [1, 2] and the network of protein fibers (50–500 nm diameter) [3–7] surrounding the cells in the microenvironment. Electrospinning is a cost-effective way of producing ultrafine fiber from wide varieties of polymers by the induction of extreme electrostatic force to a polymer solution. In particular, biocompatible nanofibrous membrane fabricated by the electrospinning process has been addressed in many literatures as a potential candidate for tissue scaffolds [3, 7–12] and drug carrier mediums [13–16]. However, apart from the biocompatibility and mechanical properties, 3D and multilayer architectures as well as the interconnected pore configuration are key structural parameters making the electrospun scaffolds convenient in tissue engineering. Nonetheless, to succeed in exploiting such 3D structural conformations, it is of significance that initially the cells introduced permeate and interact within the different depths of the scaffold profile. In other words, the cellular viability is correlated with the degree of infiltration and attachment of cells within the fibrous matrix. In both in vitro and in vivo systems, regardless of the different aspects in microfluidics of cell intrusion, the cellular diameter and corresponding structural properties have a paramount effect in determining the mechanism of cell culturing. This may be due to the fact that the mechanism of cells growth is a size-dependent phenomenon within the scaffold architecture. For instance, previous literature has reported that, for a successful attachment and permeation of bladder smooth muscle cells, the optimal pore sizes in a scaffold should be around 100–300 *μ*m [17] and for skin regeneration are 20–125 *μ*m [18] while this value for chondrocyte ingrowth is assumed to be between 70 and 120 *μ*m [19]. It is hypothesized that, for cellular size beyond optimal pore sizes, seeded cells with greater size utilize only the surface of the scaffold as an attachment site and might be forming the cellular aggregations based on cell-cell interaction. On the contrary, for cells smaller than optimal diameter, they have a tendency for revisable infiltration (migration) through the depth of scaffold profile (see Figure 1(a)). Both circumstances may reduce cell-matrix interactions, and thus negative trends for cultivation performance could be created. On this basis, the design of the customized scaffold that is able to facilitate the cellular permeation through the depth of a nanofibrous scaffold still remains a challenge in tissue regeneration [20–24]. Beyond this, the small pore sizes prevent vascularization of biomaterials leading to limitations in nutrient delivery and waste removal, resulting in disturbance to the tissue ingrowth [20, 21]. Previous works have proposed a large variety of techniques and approaches so as to tackle and compensate for this challenge. Moroni et al. [25] designed a hybrid 3D scaffold by integration of 3D macrofiber deposition with electrospun microfibers and claimed that microfibrillated networks can enhance the cells entrapment as well, increasing the cartilage tissue formation. Dubas et al. [26] modified the surface of a polycaprolactone (PCL) nanofiber scaffold by coating it with polyelectrolyte multilayer thin film. They suggested that surface coating improved the adhesion of L929 fibroblast cells in a nanofibrous scaffold. Canbolat et al. [27] proposed two approaches in order to improve permeation and dispersion of cells within the nanofibrous scaffold as well, so as to enhance the thickness of the electrospun mat. These techniques were introduced as cell electrospinning and cell layering, of which the first one completely failed due to cellular dyeing during the fiber formation mechanism. In addition, Nam et al. [28] utilized a combination of electrospinning and salt leaching processes, whereas Leong et al. [29] produced nanofibers by a cryogenic electrospinning technique so as to optimize porosity characteristics in the tissue scaffold. In other approaches, Lee et al. [30] used ultrasonication, while Baker et al. [31] produced dual-polymer nanofiber composite scaffolds from polycaprolactone (PCL) and polyethyleneoxide (PEO) by coelectrospinning, and subsequently PEO content was removed as the sacrificial fiber in order to induce higher pore size in the fibrous composite scaffolds.

Although the techniques mentioned are to some extent successful in their implementation, they reduce the simplicity and versatility of electrospinning. To retain inherent processing advantages in electrospinning, it is of the utmost importance that the methods proposed are established based on the fundamental aspects of the fiber formation mechanism in the electrospinning process. In this regard, while processing parameters and solution properties can alter the architecture and morphological characteristics of electrospun nanofibers, it is obvious that they can be utilized as a main or subsidiary technique for the structural modification in an engineered scaffold.

Chitosan as a biodegradable polysaccharide derived from partial deacetylation of chitin [33, 34] has broadly been utilized in the fibrous architecture for tissue scaffolds [35–39] and wound dressing [14, 40]. A chitosan nanofiber scaffold can reduce infection in in vivo implantation due to its antibacterial properties. It possesses better adhesion, and viability compares to its film in hepatocytes cultivation [37]. Apart from nontoxicity and the morphological similarity of chitosan nanofibers to native skin ECM, oxygen permeability, originating from its porosity characteristics, makes it appropriate for wound healing applications [14, 41]. Particularly chitosan will gradually depolymerize in to* N*-acetyl-d-glucosamine enables it to initiate fibroblast proliferation, and associates in ordered collagen deposition and stimulates the increased degree of natural hyaluronic acid synthesis in the wounded regions [14, 41, 42].

Pure chitosan is difficult to electrospin, and the mechanism of fiber formation should be facilitated by blending with cospinning polymers. The cospinning polymers can lead to higher chain entanglement, which is a main prerequisite in electrospinning for attaining nanofibers with fewer structural imperfections [43]. In addition, the composition of chitosan with other polymers may result in a higher analogy of the scaffold to natural ECM components or induces the superior properties required in tissue regeneration. Fibrous chitosan composites produced by electrospinning have been widely reported in previous papers [13, 35, 38, 39, 43–48]. For instance, chitosan/PEO nanofibers can be utilized as a three-dimensional scaffold for cartilage tissue repair due its good adhesion, proliferation, and viability for chondrocytes [48].

The aim of this work is to evaluate structural performance relations in nanofibrous scaffolds based on the establishment of a comparative analysis between simulated structural elements and cells viability. To this end, the structural characteristics are simulated by an image analysis method introduced in our previous work [32]. This method can evaluate the overall porosity and also pore size, enabling simulation of the interconnectivity and scaffold percolative efficiency (SPE) through the depth of scaffold profile [32]. The overall porosity is measured based on a projection of nanofiber structure in a 2D plane [32] whilst the interconnectivity is estimated based on the trend of blocking open channels within the depth of the scaffold profile [49]. In the present work, a chitosan/PEO polymer blend (90 : 10), due to its unique properties in biomedical applications, was electrospun, based on variation in processing parameters, so as to produce different sample scaffolds. The strong hydrogen bonding in chitosan and PEO chains leads to polymer blends being electrospinnable [50] (see Figure 1(b)). Mouse fibroblast cells (L929) were cultured in prepared scaffolds for 2 days and an MTT assay was used for assessing the cell viability and attachment.

## 2. Materials and Methods

### 2.1. Materials

Low molecular weight chitosan (*M*_W_ = 120 kD_a_) with a degree of deacetylation (DD = 75–85%) was purchased from Fluka, Switzerland. Polyethylene oxide (*M*_W_ = 900 kD_a_) was purchased from Sigma Aldrich, USA. Both glutaraldehyde solution (GA) 25 wt% in H_2_O and acetic acid (glacial) 100% were obtained from Merck Co., Germany. Mouse connective tissue fibroblast cells (L929) were obtained from the Pasteur Institute of Iran (NCBI), cultured in RPMI 1640, and maintained in an incubator with a humidified atmosphere of 5% CO_2_ at a temperature of 37°C. RPMI 1640 and trypsin were purchased from local vendors, originally made by PAA (Austria).

### 2.2. Solution Preparations

PEO was used as a cospinning component in the chitosan solution due to the difficulty of forming the continuous fibers without structural imperfections from neat chitosan in the electrospinning. Both chitosan and PEO were prepared at 2.5 wt% and dissolved solely in 90 Vol% acetic acid. Subsequently, chitosan and PEO solution in a weight fraction of 90 : 10 were mixed and stirred by mechanical mixture for 24 hours so as to make the solution homogenous.

### 2.3. Electrospinning Setup

The nozzle syringe was loaded with 5 mL of chitosan/PEO solution and the rest of the confined air in the syringe was evacuated completely. A metal capillary (needle of gauge 18, inner diameter = 0.84 mm) was inserted at the tip of nozzle. The electrospinning was performed by fully automated Electroris (Fananvaran Nano-Meghyas) equipment. The collector was covered in aluminum foil, and the anode and cathode electrodes were connected to the collector and the tip of metal capillary, respectively. Subsequently, feed rates, tip to collector distances, applied voltages, and constant rate of traverse were set by the control panel of system. Finally, the chitosan/PEO solution was electrospun based on the different processing parameters summarized in Table 1. Each sample was electrospun on the condition that only one processing parameter was changed while other parameters remained constant.

### 2.4. Cross-Linking

To prevent the dissolving of chitosan scaffolds in the culture medium as well as to provide a control on chemical and water solubility, they were cross-linked under exposure of vaporized glutaraldehyde (GA), rising from 5 mL GA solution in a desiccator, at room temperature for 48 hours.

### 2.5. Morphology of Chitosan/PEO Scaffold

The different chitosan/PEO scaffold samples were cut and gold-coated, and their morphologies were observed by field emission electron microscopy (FE-SEM), Hitachi S4160.

### 2.6. Structural Characteristics Simulation

The structural characteristics simulation and corresponding measurements were carried out based on an adaptive image analysis and local criterion that was presented in detail in our most recent work [32]. Four FE-SEM images at a magnification of 20 KX were selected from different parts of each sample scaffold and utilized as input data in the image analysis and structural reconstruction.

**Figure 2:**
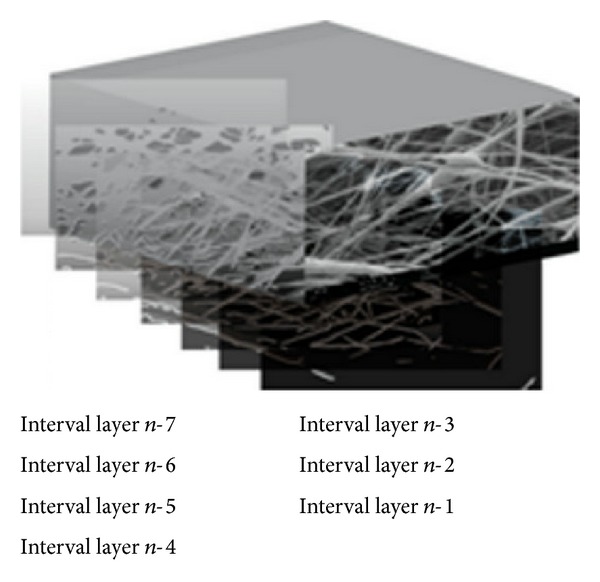
3D illustration of nanofibrous scaffold segmented by seven cumulative layers (interval layers) from infinite layers [32].

Overall porosity was measured by projection of fibrous network (solid area) in a 2D plane, while the interconnectivity was evaluated by the rate of blocking of the open channels in the depth of scaffold profile. This rate can be estimated by an increased trend of cumulative open area from sublayers to surface layers in the depth of the scaffold profile (see Figure 2) [32].

The numerical value of the blocking open area is equivalent to the layered porosity at depth *x* in a scaffold profile (*P*_*xb*_) which was measured from the following equation [32]:

(1) $$\begin{array}{c} P_{xb}=100\times \left(1-\frac{\int_{x}^{b}t\left(x\right)dx}{\int_{a}^{b}t\left(x\right)dx}\right),\;x\leq b, \end{array}$$

where *t*(*x*) is the differential histogram curve and *b* and *a*, respectively, are the supremum and infimum of a differential histogram of grayscale in an image captured from a scaffold.

To simulate a scaffold with finite layers (*n*), it is possible to select the best intervals  (INT_*n*_) exhibited in (2), where *μ*, *σ*, *b*, and *a* are defined as the mean and standard deviation and supremum and infimum of a differential histogram of grayscale, respectively [32]:

(2) INTn={∀x, x,n∈N0, x≤n:Lx=[μ±xσ,b]} ∪{[σ,μ],[a,b]}, Lx⊂[a,b].

In addition, a scaffold percolative efficiency index (or permeation efficiency constant) was defined by the following equation [32]:

(3) $$\begin{array}{c} \text{SPE}=\frac{P}{H}, \end{array}$$

where *P* is the overall porosity and *H* is the reciprocal of interconnectivity index obtained from regression analysis of layered porosity (the slope of the best-fit curve).

The pore size was estimated by the measurement of the maximum Feret diameter, which is defined as the longest distance between two points in the boundary of a pore [32]. Mean fiber diameter was evaluated by 100 measurements for each sample scaffold. The proposed simulation and measurements were implemented by ImageJ, version 1.43 m (National Institutes of Health (NIH)).

### 2.7. Cell Culturing

L929 cells were cultured in RPMI 1640 medium supplemented with 10% fetal bovine serum (FBS, Gibco, Scotland) and maintained at 37°C in a humidified atmosphere with 5% CO_2_. When cells reached >80% confluency, they were detached using 1 mL of 0.25% trypsin as mentioned previously [51].

### 2.8. Sample Preparation

Each sample scaffold was cut according to the diameter of the wells and each of them was placed into a separate well from a sterile 96-well tissue-culture polystyrene plate. Each well was seeded with 200 *μ*L of the cell suspension (10^5^ cells/mL). Empty wells were used as a control for cell attachment for a sample of the scaffold. The samples were sterilized under exposure to ultraviolet (UV) for one hour.

### 2.9. Cells Attachment and Viability (MTT Assay)

Cells were allowed to proliferate for 48 hours in the presence of scaffolds and were then rinsed with 200 *μ*L/well phosphate buffer (PBS) to remove unattached cells before adding MTT (5 mg/mL of PBS, Merck, Germany). The MTT assay is a quantitative colorimetric method based on reduction of the yellow tetrazolium dye (MTT) to purple insoluble formazan through mitochondrial succinic dehydrogenase. This method enables evaluation of the metabolically active cells, which directly reflects the cells viability. On this account, a mixture of serum free culture medium and MTT solution in fraction of 30 : 70 was added to each well. The MTT assay was performed as mentioned previously [51]. The MTT assay was repeated for each sample scaffold three times (*n* = 3).

### 2.10. Statistical Analysis

Regression analysis for finding the best-fit curve for layered porosities in structural characteristics simulation and a comparative study between simulated data obtained from image analysis and cell viability derived from in vitro experience were carried out by utilizing GraphPad Prism version 6.01 software (GraphPad Software, Inc.). All of the quantitative results were presented with mean and standard deviations. Statistical analysis was implemented, based on unpaired Student's *t*-test criteria. *P* values smaller than 0.001 were considered statistically significant which is much more reliable than *P* < 0.05, which is generally used as an upper cutoff value in statistical hypothesis tests.

## 3. Results and Discussion

**Figure 3:**
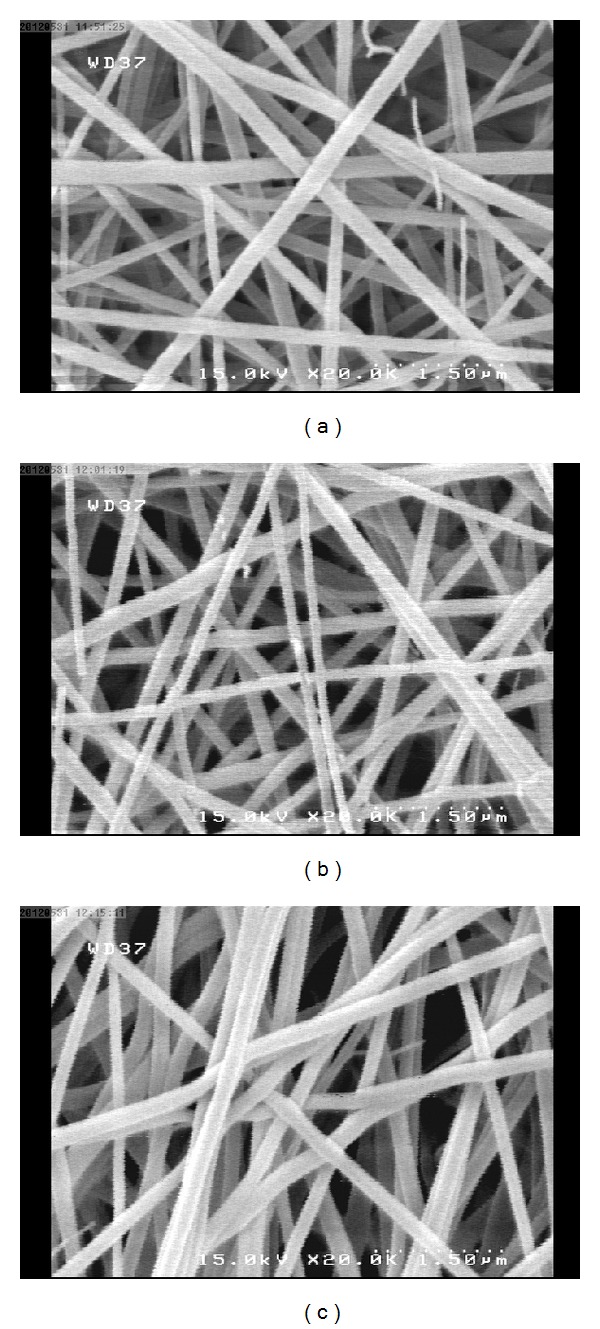
FE-SEM images in magnification of 20 KX, from nanofibrous scaffolds, (a) sample 3 (variation on deposition time), (b) sample 5 (variation on feed rate), and (c) sample 7 (variation on voltage).

**Figure 4:**
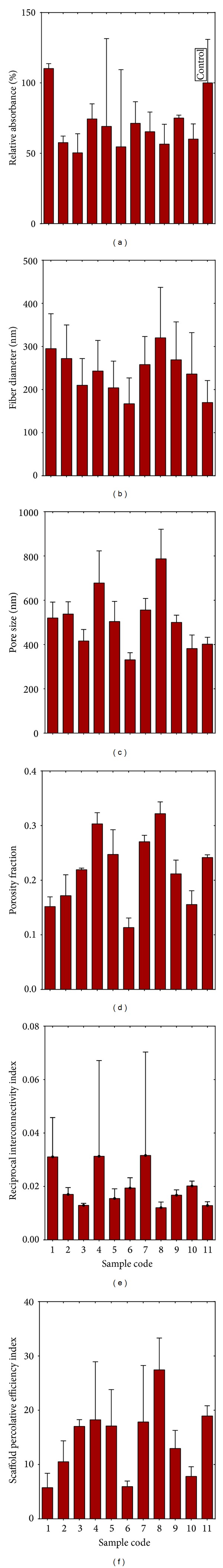
The diagrams of the mean value of relative absorbance, fiber diameter, pore size, overall porosity fraction, reciprocal interconnectivity index, and scaffold percolative efficiency for different sample scaffolds, respectively. *P* values smaller than 0.001 were considered statistically significant in unpaired Student's *t*-test.

Although attaining defect-free fibrous scaffold from electrospinning of chitosan solution is a significant challenge, the appropriate viscoelasticity of driven jet led to the formation of the smooth fibers without structural imperfections (beaded fibers and solution drippings). The morphologies of sample scaffolds after cross-linking with GA at different processing setups are shown in Figure 3. The observations of FE-SEM images revealed that there are no paramount morphological variations in different sample scaffolds that can be expressed qualitatively. On the other hand, the results obtained from MTT assay revealed that the chitosan/PEO scaffolds produced are not cytotoxic, because the mean relative absorbance and thus mean cell viability are higher than half of the value attributed to the control sample (scaffold-free well). In addition, the mean value of viable cells in different samples was enhanced from 50% to 110% compared to that belonging to the control (see Figure 4). The nanofibers constituting the 3D scaffold had a mean diameter between 170 and 320 nm, while the mean pore sizes as well as overall porosity fraction varied between 330 and 790 nm and between 0.11 and 0.32 in different samples, respectively. The differences between viable cells in the samples scaffold reflect the significant effects of structural characteristics in the mechanism of cell growth even at nanoscale. The results obtained from image analysis and simulations of structural characteristics are illustrated in Figure 4. Statistical hypothesis tests were implemented based on unpaired Student's *t*-test criteria for comparing two mean groups consisting of the mean values of relative absorbance versus mean fibers diameters, mean pores size, mean overall porosity, mean reciprocal interconnectivity index, and mean scaffold percolative efficiency separately. *P* values were obtained between relative absorbance and each structural element was smaller than 0.001 which shows that these parameters are highly statistically significant in cellular viability. Furthermore, to find the degree of correlation of each parameter with the number of viable cells, the Pearson correlation coefficients were measured. The Pearson correlation coefficient for the overall porosity and scaffold percolative efficiency were negative, which reveals that, by increasing the porosity and scaffold percolative efficiency, the number of viable cells decreased, and vice versa. On the contrary, the Pearson correlation coefficient for pore and fiber size and reciprocal interconnectivity index were positive and have an identical trend with regard to variations of viable cells. In the electrospinning process, fiber size with porosity has an opposite correlation, whereas pore size and fiber size have an identical trend [52–55]. In addition, based on our simulation [32], fiber size and reciprocal interconnectivity have a direct correlation. This means that fiber size and interconnectivity have an opposite correlation. This is due to the reduced size of fibers in the electrospun mat increasing the interconnectivity. From comparative analysis it was found that, when percolative efficiency (as a value obtained from dividing porosity on reciprocal interconnectivity, see (3)) decreased, the viability increased. Previous research has also shown that, by increasing fiber size and thus pore size in the electrospun scaffold, the cellular infiltration and viability were enhanced [52, 56, 57], and the comparative study also reflects these phenomena.

It is evident that the packed surface morphologies and small pore sizes of the electrospun membrane hinder cellular permeation and attachment, and this phenomenon leads to a reduction of viability. This is because permeation of cells in 3D architecture of scaffolds was prevented. In this situation, an adhesion site along fibers is a more beneficial parameter for cell attachment and viability. On this basis, high fiber size and consequently larger pores for maintaining an adequate attachment site for interaction between cells and scaffold matrix are an appropriate condition for achieving maximum viability. Inversely, increased porosity based on small pore size reduces interaction between cells and scaffold matrix.

To enhance the cell attachment in the 3D scaffold, it is important that cells diffuse in different layers of depth of the scaffold profile. If the scaffold has an optimal pore size, the interconnectivity of the scaffold leads to permeated cells being able to take advantage of entire scaffold layers in depth profile. However, in the case of small pore sizes, the cell introduced can only interact with the surface layers of the scaffold. In this regard, if the layers are more packed in the depth of scaffold profile, cells can take advantage of a larger area of surface layers for the attachments. Therefore, if interconnectivity decreases, the scaffold mostly exhibits 2D architecture, which is beneficial for surface cell attachment. It is because of the faster blocking of the pores in the surface layers (low interconnectivity) that they can provide a higher attachment site for cells and thus increase the viability. This phenomenon is also reflected in the statistical analysis in which reciprocal interconnectivity has an identical trend with viability, and the lower values of viability in some samples compared to control show these facts.

From the discussion above, the comparative analysis obtained from structural simulation and in vitro experiences are highly compatible with empirical and theoretical results attributed to structural elements and cellular viability in nanofibrous scaffolds. It can be concluded that the structural elements have significant impacts on the cellular attachment and viability in the nanofibrous membrane. In addition, the size of porous structures and cells determined the quality of cellular permeation and interaction in the scaffold matrix. However, this quality can be enhanced by optimizing structural elements even at nanoscale, resulting from manipulation of processing parameters in electrospinning.

To summarize, the present work can be utilized (a) to test the liability of proposed image analysis for structural characterizations of nanofibrous scaffolds for cell culturing, (b) to quantify the structural characteristics in narrow distribution of fibers diameter, while electrospinning biocompatible polymers such as chitosan, producing defect-free nanofiber scaffolds in large distribution of fibers diameter, is difficult (in most papers, the relationship between porosity, interconnectivity or fiber diameter, and cell viability has been expressed qualitatively), (c) to study the effects of processing parameters in resultant structural parameters and subsequently their effects on viability of cells, and (d) to find the optimized nanofibrous scaffolds regarding structural properties for obtaining better cell culturing performance for future work.

## 4. Conclusion

In this work, to elucidate the effects of structural characteristics on cellular infiltration and viability a comparative study was conducted between structural characteristics and cellular viability. The structural characteristics such as pore size, porosity, pore interconnectivity, and scaffold percolative efficiency were simulated by image analysis. Mouse fibroblast cells were cultured on different samples of 3D nanofibrous chitosan/PEO scaffolds produced by manipulation of processing parameters in electrospinning. The cell viability was assessed by MTT assay. The results obtained from the MTT assay showed that nanofibrous chitosan/PEO scaffolds were not cytotoxic. A comparative study revealed that the packed morphology of sample scaffolds hinders cellular infiltration and attachment. However, the mean cellular viability rose from 50 to 110% compared to that belonging to the control even at narrow distributions of mean fiber diameter and pore size from 170 to 320 nm and 330 to 790 nm, respectively. In addition, it was observed that cell attachment and viability were enhanced by increasing fiber size and pore size, whereas on the contrary this trend was the opposite for overall porosity, interconnectivity, and scaffold percolative efficiency. This may be due to increased fiber diameter and thus pore size increasing the permeation and attachment of cells, while the other parameters to some extent act inversely in electrospun scaffolds. Furthermore, the results obtained from the comparative study were highly compatible with empirical and theoretical concepts attributed to electrospun nanofibrous scaffold and cell viability in tissue engineering.

## Figures and Tables

**Table 1:** A summary of electrospinning setup and the corresponding processing parameters for each sample scaffold.

Sample code	Deposition time (h)	Feed rate (mL·h^−1^)	Applied voltage (kv)	Distance (cm)
Variation on time				
1	1	0.27	8	13
2	2	0.27	8	13
3*	3	0.27	8	13
4	4	0.27	8	13
Variation on feed rate				
5	3	0.17	8	13
6	3	0.37	8	13
Variation on voltage				
7	3	0.27	10	13
8	3	0.27	13	13
9	3	0.27	15	13
Variation on distance				
10	3	0.27	8	10
11	3	0.27	8	15

*Control sample was electrospun with constant processing parameters; deposition time is 3 h, feed rate is 0.27 mL·h^−1^, applied voltage is 8 kv, and tip to collector distance is 13 cm.
